# Case Report: Loss-of-function TRPM4 mutation p.L91Δ implicated in progressive cardiac conduction defect

**DOI:** 10.3389/fphys.2025.1681438

**Published:** 2025-10-21

**Authors:** Anne-Flore Hämmerli, Daniela Ross-Kaschitza, Prakash Arullampalam, Anna Shestak, Jimmy Jyh-Ming Juang, Nada El Makhzen, Bianca Sol Soloaga Ricciardi, Alexandre François Edmond Bokhobza, Jean-Sébastien Rougier, Elena V. Zaklyazminskaya, Jacek Gajek, Can Hasdemir, Hugues Abriel

**Affiliations:** ^1^ Institute of Biochemistry and Molecular Medicine, and Swiss National Centre of Competence in Research (NCCR) TransCure, University of Bern, Bern, Switzerland; ^2^ Department of Clinical and Preventive Genetics, Petrovsky National Research Center of Surgery, Moscow, Russia; ^3^ National Taiwan University Hospital and National Taiwan University College of Medicine, Taipei, Taiwan; ^4^ Medical Faculty, Wrocław University of Science and Technology, Wrocław, Poland; ^5^ Department of Cardiology, Ege University School of Medicine, Izmir, Türkiye

**Keywords:** TRPM4, cardiac conduction defects, inherited channelopathy, calcium-activated non-specific cation channel, loss-of-function mutation

## Abstract

**Background:**

The calcium-activated non-specific cation channel TRPM4 mediates membrane depolarization in many cell types, including cardiomyocytes and Purkinje cells. Rare genetic alterations in the *TRPM4* gene can cause familial cases of progressive cardiac conduction defects (PCCDs).

**Methods and results:**

Genetic testing was performed using whole-exome sequencing (WES). Modified human embryonic kidney cells overexpressing either wild-type or the variant p.L91Δ of human TRPM4 were used to investigate the biochemical and functional consequences of this deletion. Western blot and biotinylation experiments revealed a significant reduction in the expression of the mutant channel compared with the wild-type. Functional experiments using the patch-clamp approach demonstrated a significant decrease in TRPM4 current, consistent with the biochemical observations.

**Conclusion:**

The new TRPM4 in-frame deletion, p.L91Δ, identified in two unrelated patients with a consistent phenotype, causes a significant decrease in channel expression, leading to its loss of function in the heterologous expression system. These findings further exemplify the role of TRPM4 in genetic cardiac channelopathies.

## Introduction

Transient receptor potential melastatin-related 4 (TRPM4) is a nonselective, cation-permeable channel regulated by multiple factors, including intracellular Ca^2+^, transmembrane voltage, and signaling molecules such as ATP and PIP_2_ (phosphatidylinositol 4,5-biphosphate) (Nilius et al., 2006; Nilius et al., 2005). TRPM4 channels are widely expressed in various cells and organs, including atrial and ventricular cardiomyocytes and the sinoatrial node, and are especially enriched in the Purkinje fibers (Hof et al., 2013; Pironet et al., 2019). The physiological function of TRPM4 channels is crucial for the beneficial cardiac remodeling induced by endurance training, for a maladaptive cardiac response in long-lasting pressure overload, and for ischemia–reperfusion-related arrhythmias (Wang et al., 2018; Gueffier et al., 2017; Hu et al., 2023). Genetic alterations of the *TRPM4* gene have been linked to various cardiac rhythm and conduction disorders (premature atrial contraction, progressive atrioventricular block, different degrees of His bundle and Purkinje fiber block, Brugada syndrome, QT interval prolongation, idiopathic ventricular fibrillation, and sudden cardiac death) (Hu et al., 2023; Liu et al., 2013; Hof et al., 2017; Holmes et al., 2025). Most of the TRPM4-related cardiac phenotypes are transmitted as a dominant trait, but autosomal recessive variants are also known (Amarouch and El Hilaly, 2020; Janin et al., 2019). High-throughput sequencing has revealed >1,000 variations in the *TRPM4* gene. Still, only a few are well-characterized, and 1093 rare variants are of unknown clinical significance (ClinVar https://www.ncbi.nlm.nih.gov/clinvar date of access 18-05-2025), and their functional effects and phenotypic spectrum require further investigation.

While *TRPM4* gain-of-function mutations are assumed to slightly depolarize the plasma membrane resting potential, which may decrease the number of sodium channels available to generate a cardiac action potential, *TRPM4* loss-of-function mutations are proposed to hyperpolarize the resting membrane potential slightly, reducing the excitability of cardiac cells (Hu et al., 2023; Colquhoun et al., 1981).

This study identifies a new *TRPM4* loss-of-expression variant found in two unrelated patients with progressive conduction defects who required the implantation of permanent pacemakers.

## Patients and methods

### Patients and families

Two unrelated patients with progressive cardiac conduction defects treated with permanent pacemakers were identified during clinical genetic screening. Proband 1 (male, Turkish) is 27 years old. At the moment of genetic testing, the patient was diagnosed with a rate-dependent complete right bundle branch block and frequent premature atrial contractions. He had no structural heart disease according to transthoracic echocardiography. Proband 2 (male, Polish) is 74 years old. At the moment of genetic testing, the patient had bradycardia of unknown duration, atrioventricular and His bundle block, and permanent atrial fibrillation.

### Genetic screening

DNA samples were isolated from venous blood leukocytes using standard methods. Genetic analysis for both patients was performed using next-generation sequencing technology on the NovaSeq 6000 instrument (Illumina® platform, CA, United States). Targeted exome panel sequencing with the TrueSight One Sequencing Panel Library Enrichment Kit (Agilent, United States) was used for the Turkish proband, and whole-exome sequencing (WES) (including all genes in the Illumina Library Preparation Kit) with the SureSelect Human All Exon V7 Library Enrichment Kit (Agilent, United States) was used for the Polish proband, both following the manufacturers’ protocols. The results were compared with the reference sequence of the human *TRPM4* gene (MIM# 606936; RefSeq NM_017636.3; Ensembl gene reference: ENSG00000130529). Sequencing data were analyzed using a bioinformatic pipeline based on a customized combination of shared access programs for the Linux family of operating system distributions. Control resequencing of the genetic variants found on WES and cascade familial screening was performed using the capillary Sanger sequencing 3500 instrument (Thermo Fisher Scientific, United States) and nanopore sequencing. The pathogenicity of variants was assessed according to the ACMG (2015) guidelines. According to the Declaration of Helsinki, written informed consent was obtained before the study. The present study was conducted in compliance with the laws of Switzerland, Turkey, Russia, and Poland regarding genetic research.

### Sample preparation for nanopore sequencing approaches

Genomic DNA was extracted and purified following the manufacturer’s protocol for the Puregene Blood Kit (#158467, QIAGEN, Germantown, MD, United States). Amplification of the whole *TRPM4* gene coding and non-coding regions has been performed by long-range polymerase chain reaction (LR-PCR) by designing three overlapping primer pairs (Primer 1: *FW*: AATGATACGTGCTGGTGAGAGAGTGATGTC, *RV*: AGAGTGAATCAGATTTGCCCACTGAGAGTT; Primer 2: *FW*: GGCCAGAAGAATTTTACGGGGGAAATACAC, *RV*: GAGGCATTCTCCAGCAAAACTCAAACTTCT; and Primer 3: *FW*: CCCCTTAAATTCCCTTACCTCTCCCTTCAC, *RV*: TGCCGAGAGAGAAAAACACGCAAAAATACA), allowing the amplification of 21.3 kb, 15.1 kb, and 22.5 kb fragments, respectively. The PCR consisted of 1 µL of 100 ng/μL of genomic DNA, 12.5 µL of TaKaRa Ex-Premier™ DNA Polymerase Dye plus (cat. #RR371A), 10.5 µL of nuclease-free H_2_O, and 0.5 μL each of 10 pmol/μL forward and reverse primers. The LR-PCR samples were of a 25 μL final volume and were amplified in a thermocycling condition of 1 min at a 95 °C denaturation step, followed by 30 cycles at 98 °C for 10 s (annealing), 68 °C for 8 min (elongation), and then 5 min at 68 °C (1 cycle). A 0.4% Tris-acetate EDTA (TAE) agarose gel was used to visualize the amplified products and run at 160 V for 30 min. Images were captured using a gel imaging device (D-DiGit Gel Scanner, LI-COR Biosciences, GmbH). LR-PCR samples were purified using the AMPure XP bead Cleanup Kit (cat. #A63881), following the manufacturer’s protocol. The procedure involved measuring the LR-PCR sample volume, adding 1.8 volumes of AMPure XP beads to one volume of the sample, allowing the magnetic beads to bind to DNA fragments, and subsequently removing contaminants from the beads and DNA. Magnetic beads and DNA fragments were washed twice with 200 µL of 80% ethanol to eliminate contaminants and eluted at the end with 40 µL of elution buffer (10 mM Tris, pH 8.0; 50 mM NaCl). After DNA quantification using the NanoDrop device (Thermo Scientific™ NanoDrop OneC Spectrophotometer), LR-PCR products were immediately used for library preparation or stored at −20 °C for long-term use.

### Nanopore sequencing

LR-PCR products were pooled for each individual with the appropriate quantity (50 ng input for each PCR-amplified purified product) and then prepared to undergo sequencing using the ONT Rapid Barcoding Kit (SQK-RBK004), following the manufacturer’s instructions; the pooled barcoded library of all individuals was loaded on a flow cell (R9.4.1) and sequenced for 5 h on the MinION Mk1C sequencing device.

High-accuracy calling mode in Guppy v.3.6.0 (ONT) was used for offline base calling to convert raw data from FAST5 to FASTQ format (http://nanoporetech.com). FASTQ reads from all individuals were aligned to the human genome reference (GRCh38) using the Pomoxis’ mini_align tool (https://github.com/nanoporetech/pomoxis). Using BAM files generated from the previous step, variant calling was performed by clair3 (https://github.com/HKU-BAL/Clair3), and VCF files were then annotated using Ensembl Variant Effect Predictor (VEP) (https://www.ensembl.org/info/docs/tools/vep/index.html). The integrated genomics viewer (IGV) visualized aligned reads (https://igv.org/).

### Cell culture, biochemistry, and electrophysiology

The complete material and method are available in the Supplementary Material. In brief, HEK-293 cells were cultured and transfected with either an empty vector (pcDNA^TM^ 4/TO), human pcDNA^TM^ 4/TO-HA-TRPM4-WT (TRPM4 WT), or human pcDNA^TM^ 4/TO-HA-TRPM4-p.Leu91Δ (TRPM4 p.L91Δ). Biotinylation and Western blot assays were performed to quantify the expression levels of TRPM4 WT and TRPM4 p.L91Δ, at the cell surface and in total, respectively. In parallel, functional experiments have been performed to investigate the functional consequences of the point mutation on the TRPM4 current.

### Data availability, analyses, and statistics

Data are represented as the means ± SEM. Statistical analyses were performed using Prism7 GraphPad™ software (GraphPad by Dotmatics, San Diego, CA, United States). Student t-tests (for biochemistry) or Mann–Whitney tests (for electrophysiology) were conducted to compare two unpaired groups. *p* < 0.05 was considered significant. No multi-group comparison has been made in this study. The original contributions presented in the study are publicly available in ClinVar (NCBI) using accession number SCV005437116.2.

## Results

### Genetic findings

Mutational screening of two probands with a consistent phenotype (progressive cardiac conduction defect treated with pacemaker implantation) revealed a rare heterozygous variant chr19-49167918-TCC-(hg38) TRPM4(NM_017636.4):c.272_274del p.(Leu91del) (for the sake of brevity, hereafter referred to as p.L91Δ). This variant was classified as a variant of uncertain significance (VUS, Class III) according to ACMG (2015) criteria (Richards et al., 2015). No additional candidate genetic variant in the genes associated with inherited cardiac conditions was identified in either proband.

#### Family 1

**FIGURE 1:**
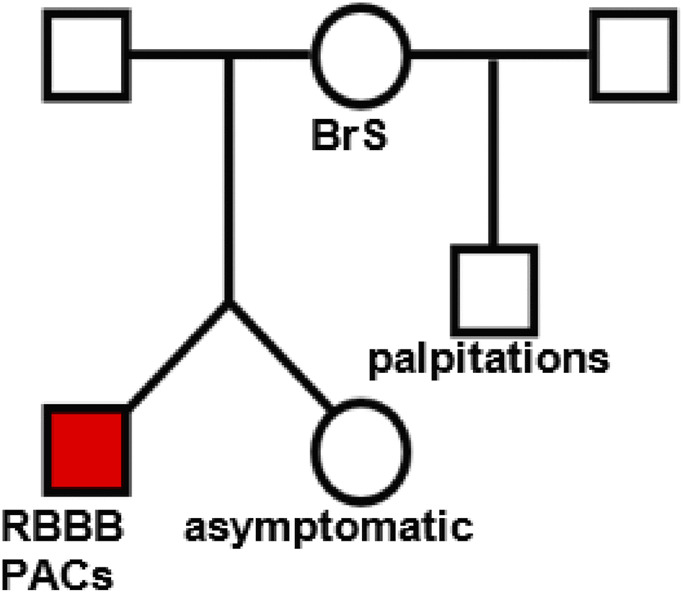
Family tree of patient 1. The proband (red square) presented with palpitations and frequent premature atrial contractions (PACs) and right bundle branch block (RBBB).

Familial cascade screening was performed (Figure 1). In parallel, using the nanopore sequencing technology, we analyzed four individuals (proband (1), parents (2), and twin sister (1)) using LR-PCR to cover the whole *TRPM4* gene coding and non-coding regions, followed by ONT sequencing. Data analysis revealed the presence of the p.L91Δ variant in the proband, his mother, and his twin sister.

The proband (red square on Figure 1), who presented with palpitations and chest pain at the age of 17, was found to have frequent premature atrial contractions (PACs). His initial PAC burden was 12.3%. His PACs spontaneously resolved during follow-up. He had rate-dependent right bundle branch block (RBBBc) at baseline. The ajmaline challenge test was “negative” for type 1 Brugada pattern (BrS). He has a structurally normal heart on transthoracic echocardiography.

The father of the proband exhibited hypertensive heart disease on transthoracic echocardiography, without evidence of a conduction disorder at baseline.

The mother of the proband exhibited hypertensive heart disease on transthoracic echocardiography, without evidence of a conduction disorder at baseline. However, she had palpitations in 2014 due to recurrent atrioventricular nodal reentry tachycardia-type supraventricular tachycardia and developed a type 1 BrS pattern with an ajmaline challenge test.

The brother of the proband does not have a conduction disorder at baseline, but mentioned palpitations without any documented evidence.

The proband’s twin sister, who is asymptomatic, exhibited no conduction disorder at baseline.

#### Family 2

In family 2, only the older brother (78 years old, phenotype-negative) was available for genetic testing. Using capillary Sanger sequencing technology with two independent pairs of primers, we were unable to detect the TRPM4 p.L91Δ variant in his DNA.

### The TRPM4 p.L91Δ variant decreases the channel expression at the cell surface

**FIGURE 2:**
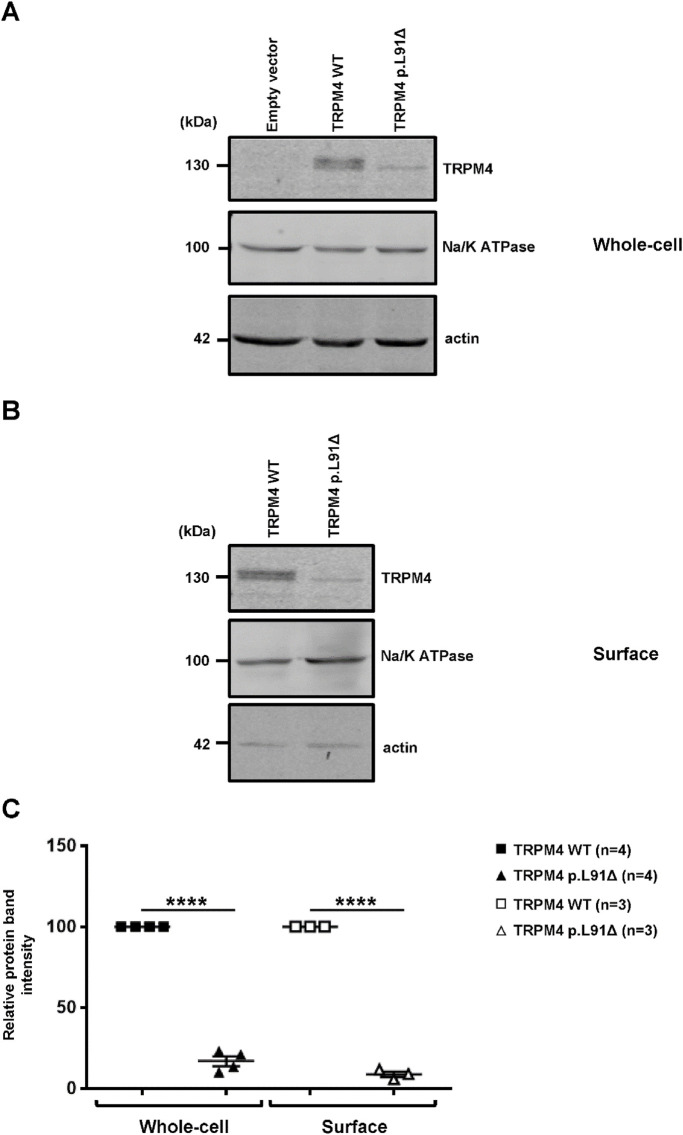
Expression of human TRPM4 wild-type and p.L91Δ variant in a heterologous expression system. **(A,B)** Western blots showing the expression of wild-type and p.L91Δ variant channels in HEK293 cells, either at the whole-cell level **(A)** or at the plasma membrane **(B)**. **(C)** Normalized dot plots of relative Western blot intensities between wild-type and TRPM4 p.L91Δ variant. **** corresponds to *p* < 0.0001 (n) as the number of replicates.

Whole-cell and plasma membrane expression experiments were performed to investigate the protein expression of this new variant using a heterologous expression system approach. Overexpressing the TRPM4 channel in HEK-293 cells, either wild-type or variant, showed that the N-terminal leucine deletion at position 91 significantly decreases protein expression at the whole-cell level (Figures 2A-C). Moreover, biotinylation experiments investigating plasma membrane expression reveal that the mutant TRPM4 p.L91Δ has a significant reduction in TRPM4 expression at the plasma membrane, confirming the loss of expression in this mutant (Figures 2B,C).

### The TRPM4 p.L91Δ variant causes loss-of-function

**FIGURE 3:**
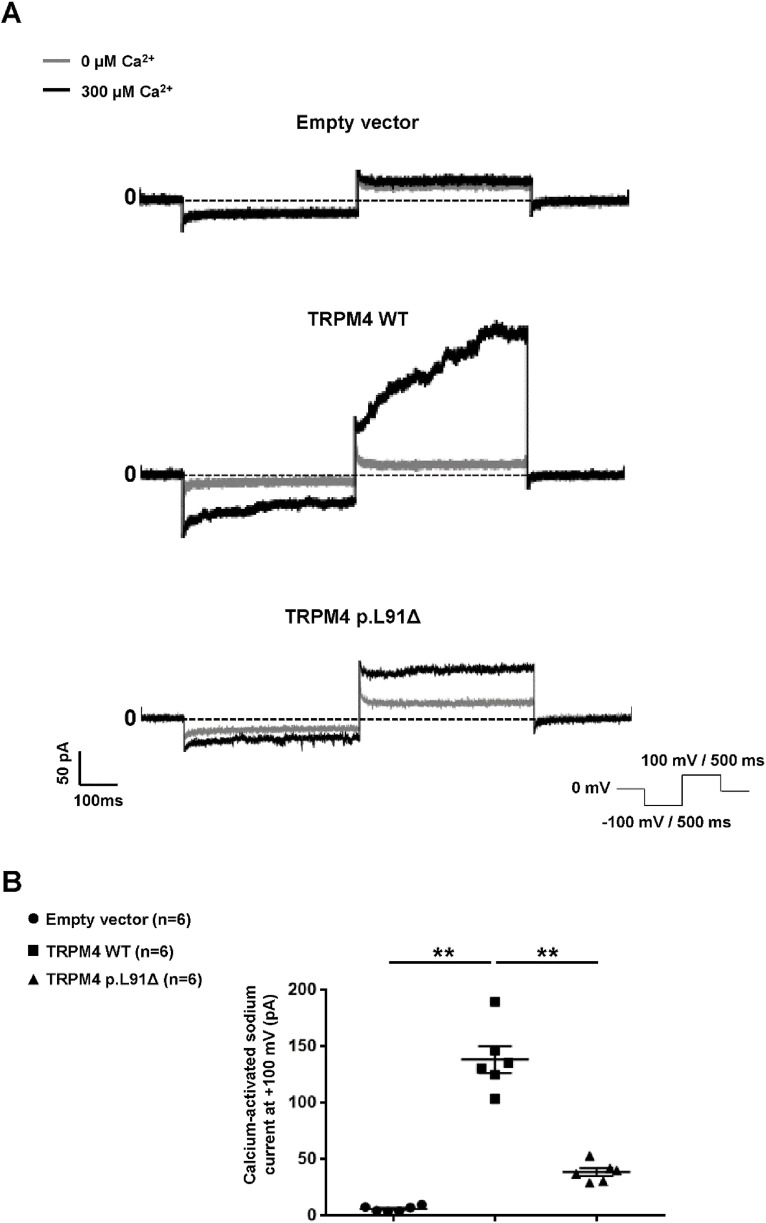
Functional characterization of human TRPM4 p.L91Δ variant. **(A)** Raw traces from HEK293 cells expressing either wild-type or p.L91Δ variant using the patch-clamp approach in inside-out configuration. **(B)** Dot blot summarizing the sodium current calcium-activated measured at +100 mV using the protocol depicted in **(A)**. ** corresponds to *p* < 0.01, and (n) is the number of cells.

Patch clamp experiments in an inside-out configuration were performed using a similar expression model to confirm the loss of function suggested by the previous results. As shown in Figure 3, the deletion of this amino acid leads to a significant decrease at depolarizing voltage (+100 mV) in the recorded calcium-activated sodium current (Figure 3B). Surprisingly, this decrease in current seems to be less pronounced at −100 mV. Calculation of the rectifying factor (current at +100 mV/current at −100 mV) for the wild-type TRPM4 and the TRPM4 p.L91Δ variant shows a significantly different result between both channels (TRPM4 wild-type: rectifying factor = −3.86 ± 0.54; n = 6 and TRPM4 p.L91Δ: rectifying factor = −0.92 ± 0.05; n = 6; *p* < 0.05). Overall, given that the background currents at −100 mV and +100 mV are similar under both conditions and that the TRPM4 current at −100 mV does not differ significantly between the channels (TRPM4 wild-type current at −100 mV = 4.68 pA ± 1.30 pA; n = 6 and TRPM4 p.L91Δ current at −100 mV = 2.15 pA ± 0.73 pA; n = 6; *p* > 0.05), these data suggest that the mutation of the leucine at position 91 mainly affects the rectifying TRPM4 current at +100 mV.

## Discussion

During the past 15 years, TRPM4 dysfunction has been linked to several cardiac conduction disorders. Although more than 1,000 variants have been reported in ClinVar, only ∼10% of them have been characterized. Among the characterized missense variants, only a few have been reported to exhibit a loss-of-function associated with reduced expression (Table 1). Interestingly, these loss-of-function TRPM4 mutants are not consistently associated with similar cardiac pathologies, suggesting a complex regulatory role of this channel in the heart (Table 1). Moreover, gain-of-function *TRPM4* pathogenic variants have already been linked to right bundle branch block; however, to our knowledge, neither premature atrial contraction nor right bundle branch block has been described as due to a loss-of-function of the TRPM4 channel (Stallmeyer et al., 2012; Liu et al., 2010).

**TABLE 1:** Rare genetic variants in the *TRPM4* gene with proven decreased protein expression in heterologous expression systems.

Amino acid alteration	Effect	Arrhythmia phenotype	Reference
p.C20S	LOF	SUD	Subbotina et al. (2018)
p.A101T	LOF	CHB	Bianchi et al. (2018)
p.A101T/P1204L	LOF	IVF	Bianchi et al. (2018)
p.P779R	LOF	BrS	Liu et al. (2013)
p.K914*	LOF	BrS	Liu et al. (2013)
p.S1044C	LOF	CHB	Bianchi et al. (2018)

The * means “stop”. LOF, loss of function; SUD, sudden unexpected death; CHB, complete heart block; IVF, idiopathic ventricular fibrillation; BrS, Brugada syndrome.

In this study, a new TRPM4 mutant, TRPM4 p.L91Δ, has been shown to causes a significant decrease in current in the heterologous expression system, primarily through the downregulation of its expression at both the whole-cell and plasma membrane levels. As observed in Figure 2, two bands are present for the TRPM4 wild-type, in contrast to the mutant, for which only the lower band is observed (Figure 2). As previously reported, this doublet is observed in heterologous expression systems and human cardiac samples due to the presence of a fully mature, glycosylated TRPM4 channel (upper band) and a partially or non-glycosylated band (lower band). The fact that only the lower band is observed with the TRPM4 p.L91Δ channel may suggest that a partially or non-glycosylated band is the only one present at the cell surface. Surprisingly, the TRPM4 channel has been shown to possess only one site of glycosylation, located on asparagine 992, which is far away from leucine 91 and raises the question, which needs to be investigated in a further study: how the mutation of leucine 91 may affect the glycosylation process of asparagine 992 (Syam et al., 2014)? The second intriguing question concerns the functional effect of this mutation. Why does this point deletion mainly affect the current at +100 mV? One explanation may reside in the fact that the TRPM4 currents recorded at −100 mV, under our conditions, are smaller than those at +100 mV. Under these circumstances, the decrease in current may seem to be more pronounced at a voltage for which the current is bigger. However, further functional experiments, beyond the scope of this case report, are needed to answer this question.

Interestingly, two missense variants affecting Leu91 (TRPM4 p.L91F and TRPM4 p.L91P) have already been reported in ClinVar (https://www.ncbi.nlm.nih.gov/clinvar/?term=TRPM4+Leu91), also associated with progressive familial heart block, highlighting the importance of leucine in position 91 for the proper structure and function of the channel.

Variant p.L91Δ is closely located to another mutation (TRPM4 p.S88A) known to affect the trafficking of this channel (Cho et al., 2014). The 3D structure of the TRPM4 protein reveals that the leucine at position 91 is part of one of the beta-sheets, comprising the amino acids 88-SNFLRLS-94, which form the MHR1 domain. These interactions with other beta-sheets are crucial for the proper folding of the protein. Deleting this leucine-91 will lead to shifting the position of the arginine-92, which contains a large, charged side chain known to interact in a non-mutated TRPM4 channel, with five amino acids belonging to other beta-sheets from the MHR1 domain (threonine-72, aspartic acid-73, alanine-74, tryptophane-191, and aspartic acid-236). Overall, this shift might destabilize those interactions, leading to improper folding. Protein misfolding is a well-known cause of intracellular degradation.

In addition, Cho et al. (2014) reported a series of experiments showing that the serine mutation at position 88 into an alanine abolished the interaction between TRPM4 and the 14-3-3γ protein. The 14-3-3γ protein belongs to the 14-3-3 family, comprising seven isoforms (β/α, γ, ε, σ, η, θ, and ζ/δ) (Obsil and Obsilova, 2011). In general, the binding of 14-3-3 modulates protein–protein interactions and the activity of their ligands (Obsil and Obsilova, 2011; Yang et al., 2006). 14-3-3 binds to the target protein, recognizing the phosphorylated (consensus sequences: R(S/X)XpSXP, RXXXpSXP, and pS/pTX1–2–COOH) and unphosphorylated motifs (Obsil and Obsilova, 2011; Yang et al., 2006). In this study, the authors demonstrated that the abolition of the interaction between the TRPM4 channel and 14-3-3γ leads to a significant decrease in the expression of this channel at the cellular surface (Cho et al., 2014). Knowing the critical role of the 14-3-3γ protein in TRPM4 regulation, it is tempting to propose that the TRPM4 p.L91Δ mutant also abolishes the interaction with this regulatory protein, partially explaining the observed results. Unfortunately, contrary to the TRPM4 p.S88A mutant, the pronounced reduction in the TRPM4 p.L91Δ mutant expression precluded co-immunoprecipitation experiments to confirm or refute this hypothesis (Cho et al., 2014).

Two unrelated probands carrying the p.L91Δ loss-of-function variant exhibit a consistent phenotype characterized by supraventricular arrhythmias (premature atrial contractions in the younger proband and atrial fibrillation in the older proband) and progressive conduction defects. However, the phenotype–genotype correlation for pathogenic variants in the *TRPM4* gene is not well understood. In contrast to other cardiac voltage-gated channels, variations in TRPM4 channels induce either gain- or loss-of-function effects, which can result in similar clinical phenotypes characterized by predominant nodal and conduction dysfunction (Palladino et al., 2022). The physiological role of TRPM4 channels in the myocardium appears to be complex and context-dependent, and likely different alterations lead to the “final common malfunction” in myocardial cells, resulting in a general phenotype (Hu et al., 2023).

The absence of a cardiac phenotype, despite the presence of the codon deletion in the *TRPM4* gene, is also emphasized in the sequencing results from different members of the Turkish family. The twin sister of the proband, carrying this gene alteration, presents no apparent cardiac dysfunctions at the age of 27 years. Still, the average age of manifestation of conduction defects in *TRPM4* pathogenic variant carriers varies significantly, generally ranging from early to late adulthood (20–71 years), suggesting that the alteration of this leucine at position 91 may lead to cardiac alterations under specific physiological conditions (Palladino et al., 2022; Bianchi et al., 2018; Daumy et al., 2016). Nevertheless, it is well known that many pathogenic variants in the genes encoding ion channels have incomplete penetrance, which can be explained by the complex nature of the conduction system and numerous intrinsic and extrinsic factors that modulate the final process (Giudicessi and Ackerman, 2013). These observations also underscore the importance of carefully characterizing new genetic variants to accurately assess their potential role in related pathologies. Conducting a family investigation is crucial for evaluating the degree of incomplete penetrance of a new variant and preventing misinterpretation and misclassification. Finally, the age-related clinical manifestation of conduction defect must be remembered during such an investigation. Cardiac dysfunction is more prevalent in aged people compared to young people. Such a cardiac disorder, observed in patients over 50 years old, may be due to overlapping causes rather than a pure alteration of TRPM4 function.

It is possible that for this gene, a significant portion of variants with changes in the primary sequence of the protein, leading to a loss of function, are related to the modulation of its expression.

## Limitations

Caution is warranted when interpreting results obtained from the heterologous expression system used in this study; the present study could not investigate the biochemical and functional consequences of wild-type and mutant TRPM4 in native physiological conditions. In addition, it may be of interest to investigate the extent to which the p.L91Δ TRPM4 channel affects the expression and function of the wild-type channel through a dominant-negative effect (whereby co-expression and interaction of the variant with the wild-type channel form a misfolded complex that is subsequently degraded, leading to a marked reduction in current) as has been reported for other homotetrameric channels (Keller et al., 2005; Chouabe et al., 1997).

## Conclusion

In a heterologous expression system, the TRPM4 p.L91Δ channel results in a loss of function, which may explain the cardiac conduction disturbance observed on the surface electrocardiogram of two unrelated patients. These findings highlight the importance of screening for the *TRPM4* gene in patients with cardiac conduction disorders.

## Data Availability

The original contributions presented in the study are publicly available in NCBI using accession number SCV005437116.1. The data (3 ppt concerning the Figure 2 and values concerning Figure 3) have been deposited on Zenodo (https://zenodo.org/) DOI: 10.5281/zenodo.17296132 https://zenodo.org/records/17296132?preview=1.
