# Supplementary material for: Case Report: Loss-of-function TRPM4 mutation p.L91Δ implicated in progressive cardiac conduction defect
Source: Front Physiol. 2025 Oct 21;16:1681438. doi: 10.3389/fphys.2025.1681438 (PMC12583068; doi:10.3389/fphys.2025.1681438)
Supplement: Supplementary file 1 [file Supplementaryfile1.docx]

**SUPPLEMENTARY INFORMATION**

**Loss-of-Function TRPM4 Mutation p.L91Δ Implicated in Progressive Cardiac Conduction Defect**

**Anne-Flore Hämmerli^1^, Daniela Ross-Kaschitza^1^, Prakash Arullampalam^1^, Anna Shestak^2^, Jimmy Jyh-Ming Juang^3^, Nada El Makhzen^1^, Bianca Sol Soloaga Ricciardi^1^, Alexandre François Edmond Bokhobza^1^, Jean-Sébastien Rougier^1*^, Elena V. Zaklyazminskaya^2^, Jacek Gajek^4^, Can Hasdemir^5^, and Hugues Abriel^1*^**

^1^ Institute of Biochemistry and Molecular Medicine, and Swiss National Centre of Competence in Research (NCCR) TransCure, University of Bern, Bühlstrasse 28, 3012, Bern, Switzerland.

^2^ Department of Clinical and Preventive Genetics, Petrovsky National Research Center of Surgery, Moscow, Russia.

^3^National Taiwan University Hospital and National Taiwan University College of Medicine, Taipei, Taiwan.

^4^ Wroclaw Medical University, Wroclaw, Poland.

^5^ Department of Cardiology, Ege University School of Medicine, Izmir, Turkey.

* Correspondence should be addressed either to:

Rougier Jean-Sébastien, PhD or Abriel Hugues, MD, PhD

Institute of Biochemistry and Molecular Medicine, University of Bern

Bühlstrasse 28, CH-3012 Bern, Switzerland

E-Mail: jean-sebastien.rougier@unibe.ch

E-Mail: hugues.abriel@unibe.ch

**Cell culture**

HEK-293 cells were cultured at 37°C in a 5% CO_2_ incubator, with Dulbecco's Modified Eagle's culture Medium (DMEM) (# 41965-039, Gibco, Basel, Switzerland) supplemented with 4 mM Glutamine, 10% FBS and 0.5% of a cocktail of streptomycin-penicillin antibiotics (10,000 U/mL, Invitrogen, Switzerland; 15140-122).

**Biochemistry**

Transfections

One hundred mm dishes (BD Falcon, Durham, North Carolina, USA) at 80% of confluence were transiently transfected using JetPEI (Polyplus transfection, Illkirch, France) following the manufacturer’s instructions. In brief, 1 µg of empty vector (pcDNA^TM^ 4/TO), human pcDNA^TM^ 4/TO -HA-TRPM4-WT (TRPM4 WT), or human pcDNA^TM^ 4/TO -HA-TRPM4-p.Leu91Δ (TRPM4 p.L91Δ) was mixed with 10 μL of JetPEI (Polyplus transfection, Illkirch, France) and 1 mL of 150 mM NaCl. After 30 min at room temperature, the cDNA solutions were applied to the cells. Forty-eight hours post-transfection, the cells were harvested for the biochemistry experiments.

Biotinylation and Western blot assays

A biotinylation assay was used to study the protein expression at the plasma membrane. In this assay, membrane proteins are labeled with biotin and immunoprecipitated with streptavidin beads to isolate the protein membrane fraction. Transfected TsA-201 cells, previously washed with cold PBS, were treated with EZlinkTM Sulfo-NHS-SS-Biotin (Thermo Scientific, Waltham, MA, USA) 0.5 mg/mL in cold PBS for 15 min at 4 °C (e.g., 2.5 mg per one hundred mm dish). Subsequently, the cells were washed twice with 200 mM glycine in cold PBS and then twice with cold PBS to inactivate and remove the excess biotin. The cells were then lysed for 1 hour at 4 °C in lysis buffer (50 mM HEPES pH 7.4, 1.5 mM MgCl_2_, 150 mM NaCl, 1 mM EGTA pH 8, 10% glycerol, 1% Triton X-100, and Complete® protease inhibitor cocktail (Roche Diagnostics, Mannheim, Germany)). After centrifugation at 4 °C, 16’000 g for 15 min, the pellet was discarded. Protein concentrations of each lysate sample were measured in triplicate by Bradford assay and interpolated by a bovine serum albumin (BSA) standard curve. Two milligrams of the supernatant were incubated with 50 μl streptavidin Sepharose high-performance beads (GE Healthcare, Uppsala, Sweden) for 2 hours at 4 °C. The beads were subsequently washed five times with lysis buffer. At the same time, 60 μg of protein was kept for loading the total protein expression. Both types of samples (biotinylated and total proteins) were incubated with 2X NuPAGE sample buffer (Invitrogen, Carlsbad, CA, USA) containing dithiothreitol (DTT) to have a final protein concentration of 1μg/μl in 1X NuPAGE sample buffer and 100 mM of DTT. Those samples were heated at 37 °C for 30 min before loading on an SDS-PAGE gel. Sixty µg of protein for the input (whole cell protein content), and half of the biotinylated fractions for each sample were loaded and run at 150 V for 1 hour on 9% polyacrylamide gels. The Turbo Blot dry blot system (Biorad, Hercules, CA, USA) was used to transfer the samples to a nitrocellulose membrane. All membranes were stained with Ponceau reagent as a qualitative check for equivalent total protein loading. Membranes were then rinsed twice with PBS and blocked with 5% BSA in PBS for 1 hour. After this blocking step, the membranes were incubated for 2 hours with rabbit anti-human TRPM4 antibody (epitope: 1137-CRDKRESDSERLKRTSQKV-1155, Pineda, Berlin, Germany) diluted 1:1’000 in PBS + 0.1% Tween, rabbit anti-actin antibody (Sigma A-2066) diluted 1:1’000 in PBS + 0.1% Tween, and mouse anti-Na^+^/K^+^ ATPase antibody (Abcam ab 7671) diluted 1:1’500 in PBS + 0.1% Tween. The membranes were washed 4 times in PBS 1X + 0.1% Tween before incubating with fluorescent secondary antibodies. Secondary antibodies IR Dye 800 CW, anti-rabbit diluted (1:15’000) in PBS + 0.1% Tween, and IR Dye 700 CW, anti-mouse diluted (1:15’000) in PBS + 0.1% Tween (LI-COR Biosciences, Lincoln, NE, USA) were added for 1 hour. After 4 washes with PBS + 0.1% Tween and 3 washes in PBS, membranes were scanned with the FUSION FX Spectra® Infrared Imaging System (VILBER smart imaging, Marne-la-Vallée, France) to detect fluorescent protein. Subsequent quantitative analysis of protein content was achieved by measuring and comparing band densities (equivalent to fluorescence intensities of the bands) using the Evolution*-*Capt software (VILBER smart imaging, Marne-la-Vallée, France)*.* The background was first subtracted for each band (human TRPM4, actin, and Na^+^/K^+^ ATPase), then TRPM4 intensity was divided by the intensity of the Na^+^/K^+^ ATPase band (for a given sample) and normalized for comparison.

**Electrophysiology​**

Transfections​

Thirty-five mm dishes (BD Falcon, Durham, North Carolina, USA) at 80% of confluency were transiently transfected using JetPEI^TM^ transfection reagent (Polyplus transfection, Illkirch, France) and following the instructions of the manufacturer. In brief, 250 ng of pcDNA^TM^ 4/TO (empty vector), human pcDNA^TM^ 4/TO -HA-TRPM4-WT (TRPM4 WT), or human pcDNA^TM^ 4/TO -HA-TRPM4-p.Leu91Δ (TRPM4 p.L91Δ) was mixed with 100 ng of a reporter gene coding for GFP. Expression of GFP was used to identify transfected cells during patch clamp experiments.​ Plasmids were mixed with 3 μl of JetPEI^TM^ and 46 μl of 150 mM NaCl. After 15 min at room temperature, the solutions were applied to the cells. Twenty-four hours post-transfection, the cells were plated at low density in a new 35 mm dish coated with poly-L-lysine. The cells were patched 48 hours post-plating (96 hours post-transfection).

Inside-out patch clamp​

Electrophysiological recordings were performed in the inside-out patch-clamp configuration with patch pipettes (1 and 2 μm tip opening) pulled from 1.5 mm borosilicate glass capillaries (WPI GmbH, Friedberg, Germany) using micropipette puller P 97 (Sutter Instruments, Novato, CA, United States). The tips were polished to achieve a pipette resistance of 2–4 MΩ in the bath solution. The pipette solution contained 150 mM NaCl, 10 mM HEPES, and 2 mM CaCl_2_ (pH 7.4 with NaOH). The initial bath solution with 0 calcium contained 150 mM NaCl, 10 mM HEPES, and 2 mM HEDTA (pH 7.4 with NaOH). After reaching the inside-out configuration, different solutions were perfused at the intracellular side of the membrane patch using a modified rapid solution exchanger (Perfusion Fast-Step SF-77B; Warner Instruments Corp. CT, United States). The first solution, applied for 3 to 5 sweeps, is the bath solution (150 mM NaCl, 10 mM HEPES, and 2 mM HEDTA; pH 7.4 with NaOH) with 0 calcium. Then, to activate TRPM4 channels, a solution containing 150 mM NaCl, 10 mM HEPES, and  300 µM Ca^2+^ (pH 7.4 with NaOH) was applied. Finally, the bath solution with 0 calcium was used to quantify the leak current. The human TRPM4 sodium currents were recorded with a Multiclamp 700B amplifier (Molecular Devices, Sunnyvale CA, United States) controlled by Clampex 10 via Digidata 1332A (Molecular Devices, Sunnyvale, CA, United States). Data were low-pass filtered at 5 kHz and sampled at 10 kHz. Experiments were performed at room temperature (23°C). The holding potential was 0 mV. The stimulation protocol consisted of two sweeps for measuring steady-state currents at a frequency of 1 Hz. The first sweep was at −100 mV for 500 ms, and the second was at +100 mV for 500 ms. The sodium currents that are calcium-activated have been calculated by averaging the last 100 ms of the second sweep at +100 mV.
